# Mechanisms of Pericyte‐Mediated Cancer Metastasis

**DOI:** 10.1002/advs.202600031

**Published:** 2026-05-15

**Authors:** Ziheng Guo, Yihai Cao

**Affiliations:** ^1^ Department of Pancreatic Surgery West China Hospital Sichuan University Chengdu China; ^2^ Department of Microbiology Tumor and Cell Biology Karolinska Institute Stockholm Sweden; ^3^ School of Pharmaceutical Science Wenzhou Medical University Zhejiang Provice China

**Keywords:** cancer, metastasis, Pericyte, tumor microenvironment, tumor vasculature

## Abstract

Emerging experimental evidence shows that perivascular cells (PCs), referred to as pericytes lying within the basement membrane (BM) and surrounding endothelial cells (ECs) of microvasculatures, play multifarious roles in cancer metastasis. In the tumor microenvironment (TME), PC detachment and ablation from tumor microvessels obliterate the vascular integrity and the protective barrier of the vessel wall, leading to increased permeability for cancer cell intravasation. PCs retain mesenchymal stem cell (MSC) features and commit to differentiation into other stromal cells, including the pericyte‐fibroblast transition (PFT) for promoting cancer metastasis. Activated PCs produce a myriad of growth factors, cytokines, and chemokines, which promote invasiveness and dissemination of cancer cells by altering tumor angiogenic, inflammatory, and immune microenvironment. Owing to their high lineage and phenotypic plasticity, PCs significantly constitute to the pre‐metastatic niche formation in distal organs. This review provides an updated overview and mechanistic insights into each of PC‐mediated critical processes of the metastatic cascade.

## Graphical abstract

Pericytes play multifarious roles in promoting cancer metastasis by facilitating metastatic cancer cell dissemination, circulating tumor cell survival, tissue extravasation, premetastatic niche formation, and metastatic tumor growth. These cells have intertwined interactions with vascular endothelial cells to modulate cancer cell behavior and invasiveness. This article provides in‐depth mechanistic insights into pericyte‐mediated distinct steps of the metastatic cascade.

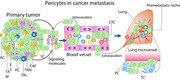

**Abbreviations**
Ang1: Angiopoietin 1
Ang2: Angiopoietin 2
ALI: Acute lung injury
α‐SMA: Alpha‐smooth muscle actin
BBB: Blood‐brain barrier
BM: Basement membrane
CAF: Cancer‐associated fibroblast
CCL: chemokine ligand
CD31: Alanyl aminopeptidase
CNS: Central nervous system
CRC: Colorectal cancer
CTC: Circulating tumor cells
CXCL: Chemokine (C‐X‐C motif) ligand
CXCR: Chemokine (C‐X‐C motif) receptor
DLK1: Delta‐like 1 homolog
EC: Endothelial cell
ECM: Extracellular matrix
EMT: Epithelial‐to‐mesenchymal transition
FOXC2: Forkhead box C2
GBM: Glioblastoma multiforme
HB‐EGF: Heparin‐binding‐epidermal growth factor
HCC: Hepatocellular carcinoma
HGF: Hepatocyte growth factor
HIF‐1α: Hypoxia‐inducuble factor‐1alpha
IL: Interleukin
KLF4: Kruppel‐like factor 4
Lyve‐1: Hyaluronic acid receptor 1
MDSC: Myeloid‐derived suppressive cell
MMP: Metalloproteinases
MSC: Mesenchymal stem cell
NG2: Chondroitin sulfate proteoglycan 4
NK: Natural killer
NNMT: Nicotinamide N‐methyltransferase
NO‐sGC: Nitric oxide‐soluble guanylate cyclase
NSCLC: Non‐small cell lung carcinoma
PC: Pericyte
PDAC: Pancreatic ductal adenocarcinoma
PFT: Pericyte‐to‐fibroblast transition
PDGF‐BB: Platelet‐derived growth factor‐BB
PDGFR‐β: Platelet‐derived growth factor‐beta
RGS5: Regulator of G protein signaling 5
ScRNAseq: Single‐cell RNA sequencing
SDF‐1: Stromal cell‐derived factor‐1
SUR2: ATP‐binding cassette subfamily C
TAM: Tumor‐associated microphage
TGFβ: Transforming growth factor beta
TIMP‐1: Tissue inhibitor of metalloproteinases 1
TME: Tumor microenvironment
VEGF: Vascular endothelial growth factor
VEGFR: VEGF receptor

## 1 Introduction

Endothelial cells (ECs) and pericytes (PCs) are two major cell types that constitute the microvessel wall [1, 2, 3]. While diverse functions of microvascular ECs in embryonic development, tissue growth, homeostasis, reproduction, aging, wound repair, and various pathological processes are relatively well studied [4, 5], the biological functions of PCs in the regulation of diverse physiological and pathological processes remain elusive. The broad definition of PC refers to mesenchymal cells that are located to the peri‐EC region of microvessels [1, 3]. However, the stringently accepted PC definition designates the perivascular cells that are embedded within the microvascular basement membrane (BM) [1]. Owing to the existence of mosaic PC populations, lack of a specific marker to distinguish PCs from other mesenchymal cells, and lack of complete BM in the tumor vasculature, PCs in this review article designate the broad term of PCs that surround microvessels regardless of their relation to BM.

Cancer cells, together with other stromal cells, including inflammatory cells, fibroblasts, vessel‐wall cells, adipocytes, nerve cells, and other immune cells constitute the tumor microenvironment (TME) [6]. Growing tumors contain an exceptionally high density of microvessels that are often partly coated by chondroitin sulfate proteoglycan 4^+^ (NG2^+^) and platelet‐derived growth factor‐beta (PDGFR‐β)^+^ PCs [7, 8, 9]. Owing to diverse tumor types, genetic mutations in cancer cells, and TME variations, the content of PCs differs significantly between tumors. It is likely that the PC content is largely pre‐determined by the source of preexisting vasculatures within the primary tumor‐originated tissues. In some tissues, such as liver and bone marrow, sinusoidal microvessels constitute the microvascular networks, which often lack PC coverage [1, 10]. In endocrine organs such as the thyroid, pancreatic beta‐islets, and adrenal glands, microvessels contain specialized ECs with high numbers of fenestrae and fewer PCs [11, 12, 13, 14]. Complex mechanisms are likely involved in the regulation of the PC‐EC interactions due to the existence of high EC and PC heterogeneity, variations of signaling pathways, and differential expression of cell adhesion molecules in various tissues and organs [15, 16].

Some PCs possess mesenchymal stem cell (MSC) and progenitor cell features, allowing them to differentiate into other cell types, including stromal fibroblasts, myocytes, adipocytes, and osteocytes [9, 14, 17, 18, 19]. Although sharing similarities with MSCs, PCs exhibit broader plasticity than MSCs for differentiation into other cell types. For example, PCs in cell culture display a more immature phenotype and broader differentiation capacity than standard MSCs, indicating a higher order of multipotency [20]. Similarly, isolated PCs exhibit more robust osteogenic differentiation relative to MSC populations [21]. Emerging evidence shows that PCs in TME can give rise to other stromal cell types such as fibroblasts and myofibroblasts, which markedly promote cancer metastasis [9]. PCs and PC‐derived other stromal cells produce a myriad of growth factors and cytokines that modulate tumor growth, invasion, metastasis, and anticancer drug responses. For example, PC‐derived Ang‐1 stabilizes the vascular wall and protects ECs from anti‐angiogenic therapies, contributing to antiangiogenic drug resistance [22]. PC‐derived‐thrombospondin‐1(TSP‐1) confers drug resistance of BRAF inhibitors and tyrosine kinase inhibitors (TKIs) by activation of a pro‐survival pathway [23]. Thus, PCs may significantly modulate TME and tumor cell behavior, which have profound impacts on cancer progression and therapeutic outcomes.

Since PCs are defined as perivascular cells, their intimate interactions with vascular ECs are essential for maintenance of their identity [24, 25, 26]. Specific signaling molecules from ECs and PCs operate microvascular PC attachment and separation [24, 25, 26]. Vascular ECs are the primary cell type that preserves the PC stemness features [26, 27, 28]. Once becoming disassociated from microvessels, PCs often loss their stemness properties and give rise to other cell types [9]. The cellular source of chemoattractive signals is probably one of the key determinants for regulating the PC‐EC interaction. For example, tumor cell‐derived PC chemotactic factors such as PDGF‐BB deter the PC‐EC interaction and ablate PCs from tumor microvessels [29].

### 1.1 Pericyte Biology

**FIGURE 1:**
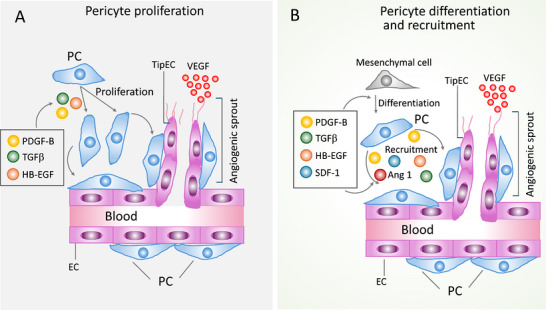
Pericyte proliferation, differentiation, and vascular recruitment. (A) Pericyte proliferation is regulated by several growth factor‐receptor pathways, including the PDGF‐B‐PDGFRβ, HB‐EGF‐ErbB, and TGFβ‐TGFβR signaling. (B) Pericytes are originated from mesenchymal cells. Multiple growth factors, including PDGF‐B, HB‐EGF, Ang‐1, SDF‐1, and TGFβ participate in recruitment of pericytes onto the microvessels. VEGF induces vascular sprouting through the VEGFR2 signaling. PDGF‐B = platelet‐derived growth factor B; HB‐EGF = heparin‐binding epidermal growth factor; TGFβ = transforming growth factorβ; Ang1 = Angiopoietin 1; SDF‐1 = stromal cell‐derived factor 1; EC = endothelial cell; PC = pericyte.

***Origin***: PCs universally exist in nearly all microvasculatures [30]. It is generally accepted that PCs in most organs, including the lung, intestine, stomach, heart, and liver are originated from the mesothelium, which shares a close ontogenic relation with stromal fibroblasts [1]. Mesothelial cells likely endure the mesothelial‐to‐mesenchymal transition (MMT) for the generation of PCs and fibroblasts [1, 31, 32]. It appears that during embryonic development PCs located in various tissues have different origins. For example, the ectoderm‐derived neural crest contributes to the PC lineage commitment in neuronal tissues and thymus [1, 33, 34]. Mural cells in the gut are derived from the mesothelium surrounding the coelomic cavities [16]. The epicardial mesothelium generates coronary pericytes [35, 36]. In physiologically and pathologically angiogenic microvessels, vascular coverage by PCs is achieved by possibly three key processes, i.e., proliferation, differentiation, and recruitment (Figure 1). Along with the formation of angiogenic vessels, these three processes occur simultaneously to stabilize the newly formed microvessels.

***Cellular markers***: Defining a specific molecular marker for all PCs remains challenging. At the time of this writing, no single specific molecular marker is available for defining PCs in various tissues. The most accepted molecular markers include PDGFR‐β, NG2, alpha‐smooth muscle actin (α‐SMA), desmin, and alanyl aminopeptidase (CD13) [1, 37]. Other molecular markers, including endosialin, potassium inwardly rectifying channel, subfamily J, member 6 (kir6.1), regulator of G protein signaling 5 (RGS5), ATP‐binding cassette subfamily C (SUR2), and delta‐like 1 homolog (DLK1) need further validation in the context of cell morphology and their relations to vascular ECs [1, 37]. Emerging new PC molecular markers include Gli1 [16, 38, 39, 40] and Tbx18 [41, 42, 43]. It should be emphasized that expression of these PC molecular markers likely experiences continuous alterations during embryonic development, adult physiology, and pathological processes, and identification of their existence often requires combinations of several markers.

***Heterogeneity***: PC heterogeneity is defined by their tissue distribution, morphology, molecular markers, and biological functions. In vivo lineage genetic tracing that tracks ECs, hematopoietic cells, and neurocrests demonstrates the existence of a high‐degree of PC heterogeneity [16, 44]. Bone marrow‐derived myeloid progenitors also contribute to certain PC generation. A PC subpopulation in the developing brain and skin tissues has the hematopoietic myeloid origin [45]. As vascular ECs and hematopoietic cells share a common precursor, i.e., the hemagioblast [46], the hematopoiesis‐derived PCs further increase complexity in understanding their interactions and heterogeneity. Even within the same tissue, PCs exhibit heterogeneous features of distribution, molecular markers, morphology, and biological functions [16]. For example, two distinct PC populations are identified in the adult skeletal muscles, which differ in their differentiation potentials into various cell types [47, 48, 49, 50]. Similarly, two bone marrow PC populations have been identified depending on their distributions in close association with arterioles and sinusoidal microvessels [51]. Along with implications of new technologies, including single‐cell‐RNA sequencing, transcriptomics, and metabolomics, new subpopulations and unprecedented heterogeneity of PCs will be expectedly emerged.

***Signaling molecules and pericyte‐endothelial cell interaction***: EC‐derived growth factors, cytokines, chemokines, and extracellular matrix (ECM) proteins are termed angiocrine signaling that acts through autocrine, paracrine, and endocrine mechanisms to control embryonic development, homeostasis, inflammation, immunity, and tissue/organ regeneration [52]. Pericrine refers to as PC‐derived signaling, including growth factors, cytokines, chemokines, and ECM proteins, which have broad functional impacts on a variety of physiological and pathological processes, including wound healing, inflammation, immunity, angiogenesis, vascular homeostasis, and metabolism [52]. Recruitment of PCs to the newly formed vasculature entails their intimate interactions with vascular ECs. Manifold signaling molecules cooperatively determine the PC coverage in microvessels, where EC and PC association and detachment relentlessly occur within a given tissue [24, 25, 26, 28]. Under physiological conditions such as wound healing and female reproductive tissues, the formation of new blood vessels is escorted by vascular stabilization and remodeling in which PCs play crucial roles in establishing a vascular network with appropriate functions [53, 54]. Similarly, angiogenesis in pathological tissues such as tumors and inflamed tissues often evokes activation of signaling pathways for PC proliferation and activation [55, 56, 57]. For example, in a mouse acute lung injury (ALI) model, the crosstalk between endothelial nitric oxide and pericyte soluble guanylate cyclase (NO‐sGC) promotes vascular integrity and prevents vascular permeability [58].

**FIGURE 2:**
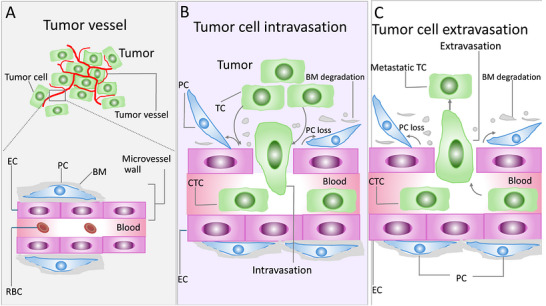
Pericytes in metastatic tumor cell intravasation and extravasation. (A) The tumor microvessel wall usually consists of three barriers, including the endothelium layer, perivascular cell layer, and the basement membrane. (B) Intravasation of metastatic tumor cells across the vessel wall entails degradation of the basement membrane, ablation of pericytes, and transmigration across the endothelium. Typically, metastatic cancer cells transmigrate through the opening of the inter‐endothelial cell tight junctions, which are tightly linked by specific adhesion molecules. (C) Extravasation of the circulating tumor cells across the vessel wall occurs in a reverse sequential event of intravasation. Circulating tumor cells needs to identify the extravasation location of endothelium in the distant organ. After opening the inter‐endothelial cell junctions, circulating tumor cells must break through the pericyte and basement membrane layers for colonization. EC = endothelial cell; PC = pericyte; BM = basement membrane; TC = tumor cell; CTC = circulating tumor cell; RBC = red blood cell.

Recruitment of PCs to the newly formed vasculatures demands intimate and intertwined signaling coordination between ECs and PCs (Figure 2). Vascular endothelial growth factor (VEGF) as the one of the most commonly available and potent angiogenic factors, primarily acts on vascular ECs through its specific VEGF receptors (VEGFRs) [59, 60, 61]. Along the formation of vascular sprouts, angiogenic ECs secrete signaling molecules such as PDGF‐B to recruit PCs through activation of the PDGFR‐β signaling [62, 63, 64, 65]. Similarly, transforming growth factor beta (TGFβ), heparin‐binding‐epidermal growth factor (HB‐EGF), stromal cell‐derived factor‐1 (SDF‐1), and angiopoietin 1 (Ang1) are also involved in PC proliferation, migration, and recruitment [1, 66, 67, 68, 69, 70]. Concomitantly, the stalk ECs express Dll4/Notch molecules to prevent the formation of excessive vascular sprouts [71, 72, 73, 74]. Other signaling molecules, such as TGFβ may coordinate the PC supply by regulating their growth and differentiation [75, 76]. Even within the same growth factor family, different members may have opposing biological functions in recruitment and ablation of PCs. For example, Ang1 and Ang2 within the angiopoietin family bind to the same Tie2 receptor in ECs but they exert opposing functions. While Ang1 promotes vascular coverage of PCs, Ang2 ablates them from microvessels [77, 78, 79]. Tie2‐expressing PCs constrain angiogenic sprouting, and specific deletion of Tie2 in PCs results in a proangiogenic phenotype and accelerated tumor growth [80]. These highly regulated and synchronized interactions between various signaling pathways in ECs and PCs befall throughout the entire processes of angiogenesis and vascular remodeling.

**FIGURE 3:**
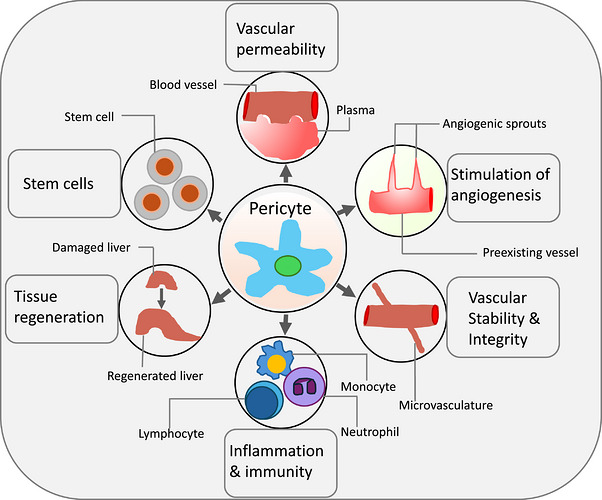
Biological functions of pericytes. Vascular coverage with pericytes protects microvessels from leakiness, regulates blood perfusion, and maintains vascular stability and integrity. In the central nervous system, pericytes significantly constitute the blood‐brain barrier against passive permeability of circulating molecules and cells. Pericytes modulate inflammation and immunity by producing growth factors, cytokines, and chemokines. They coordinately interact with vascular endothelial cells to regulate immune and inflammatory cell adhesion, rolling, migration, and crossing the microvessel wall. Pericytes also possess stem cell features and differentiate into other cell types to actively participate in tissue/organ regeneration.

***Physiological functions***: PCs are pluripotent cells that display multifarious biological functions in maintenance of vascular homeostasis and regulation of microvascular perfusion and permeability (Figure 3). These cells possess stem cell features and can differentiate into various cell types during embryonic development and tissue/organ regeneration. PCs also actively participate in the regulation of inflammatory response and immunity by producing various signaling molecules, including growth factors, cytokines, and chemokines [81, 82]. Transmigration of inflammatory cells and immune cells through the microvessel wall must cross the PC barrier for extravasation. Numerous studies demonstrate that the vascular structure and penetrability are key determinants for recruitment of inflammatory and immune cells in both healthy and pathological tissues [83]. PC loss in microvessels permits exposure of angiogenic stimuli to ECs and subsequent angiogenesis. Conversely, vascular coverage with PCs prevents excessive angiogenesis and vascular leakiness [84, 85]. In some neural tissues, such as retina, microvessels are tightly covered by PCs to prevent leakiness [86]. Owing to lack of lymphatics in the retinal tissue for the collection of extravasated fluid, the compact coverage of retinal microvessels with PCs provides an efficient mechanism against leakiness. In the central nervous system (CNS), PCs play a crucial role in the constitution and maintenance of blood‐brain barrier (BBB), which prevents passive transport of cells and molecules from the blood stream [87, 88]. PCs also control microvascular perfusion by regulating capillary contraction and dilation [89]. Together, PCs display multiple functions in controlling microcirculation, and their dysfunction would lead to the onset and development of vascular diseases.

### 1.2 Dysregulation of Pericyte‐Endothelial Cell Interactions in Tumors

Interactions between PCs and ECs are mediated by intercellular junctions, including gap junctions, adhesive plaques, and peg‐and‐socket junctions [82]. Aberrant expression of these intercellular junction molecules in vascular cells often occurs in various tumor vessels, leading to frequent dysregulation of the PC‐EC interactions [90]. Owing to genetic and epigenetic alterations and metabolic reprogramming, solid tumor tissues often produce imbalanced levels of various angiogenic and vascular remodeling signaling molecules. For example, VEGF expression levels are often elevated in tumors owing to tumor genetic mutations of cancer cells, hypoxia, infiltration of stromal cells such as fibroblasts, and inflammatory cells [91]. Genetic mutations of KRAS oncogene and the p53 tumor suppressor gene in malignant cells markedly augment VEGF expression levels [92, 93, 94, 95]. Epithelial cell‐originated cancers with KRAS mutations, such as pancreatic ductal adenocarcinoma (PDAC), hepatocellular carcinoma (HCC), colorectal cancer (CRC), and non‐small cell lung carcinoma (NSCLC) often lack PCs in their microvasculatures [92, 96]. Functional activation of KRAS by mutations elevates expression levels of Forkhead Box C2 (FOXC2), which directly targets the promoter of angiopoietin 2 (Ang2) for upregulation [92]. The elevated Ang2 expression ablates the PC content from the tumor microvessels.

Rapid‐growing tumors inevitably experience tissue hypoxia that induces VEGF expression through the HIF‐1α‐mediated transcriptional regulation [97, 98]. VEGF potently augments tumor angiogenesis by the mechanisms of stimulating the tip‐EC formation and EC proliferation and migration [99]. VEGF also potently stimulates vascular permeability by the formation of the primitive vascular networks, which often lack complete BM and PC coverage [86, 100, 101]. In other experimental settings, such as the retinal vasculature, VEGF has been reported to ablate PCs from microvessels [86]. Hypoxia, together with anaerobic metabolism‐augmented acidosis differentially regulates expression levels of various vascular factors [102]. While VEGF levels are markedly elevated in most tumors, PDGF‐B, Ang‐2, and TGFβ production are disproportionate to match high VEGF production. Consequently, the tumor vasculature manifests disorganized phenotypes and leakiness by lacking PC coverage [103]. PC focal adhesion kinase (FAK) inhibits angiogenesis and tumor growth through negative regulation of the Gas6/Axl signaling [104]. PCs modulate tumor growth through the β3‐integrin‐mediated paracrine signaling, including the FAK‐p‐HGFR‐p‐Akt‐p‐p65 signaling, chemokine (C‐X‐C motif) ligand1 (CXCL1), chemokine ligand 2 (CCL2), and tissue inhibitor of metalloproteinases 1 (TIMP‐1) [105].

The cellular source of PC‐signaling molecules plays a critical role in the regulation of PC coverage in tumor microvessels. Although PDGF‐BB generally promotes PC coverage in newly formed microvessels [64], the tumor cell‐derived PDGF‐BB can also provoke PC loss [29]. Similar to the VEGF scenario, tumor cell‐derived PDGF‐BB molecules build a chemoattractant gradient around tumor cells [106], resulting in vascular PC loss by stimulating their migration toward cancer cells. These findings have been implicated in cancer therapy by defining PDGF‐B^+^ tumors as a preferred target for anti‐PDGF therapy [29]. In preclinical cancer models, pharmacological inhibition of the PDGFR‐β signaling by drugs such as imatinib potentially promotes cancer metastasis by ablation of vascular PCs [29].

### 1.3 Remodeling of the Pericyte‐Immune Cell‐Cancer Cell Network

In TEM, PCs have intertwined interactions with both inflammatory and immune cells by modulating their transmigration through the vessel wall and immune suppression [107]. Activated PCs in tumors produce proinflammatory cytokines to recruit macrophages and phenotypically convert them into the M2‐like tumor‐associated microphages (TAMs) [108]. Inhibition of the Ang2‐Tie2 signaling promotes M1‐TAM recruitment and reduces M2‐TAM and Treg cell infiltration [109]. PDGF‐C^+^TAMs stimulate PDGFRα^+^PC proliferation [107, 110]. In a mouse melanoma model, specific deletion of NG2 in myeloid cells reduces TAM recruitment by impairing PC‐EC interactions [111]. In a mouse GBM model, M2‐TAMs markedly promote PC recruitment and migration [112].

Several studies report that immunosuppressive functions of PCs in tumors. For example, GBM PCs acquire immunosuppressive and anti‐inflammatory phenotypes by producing various cytokines, including IL‐1, IL‐6, IL‐10, IL‐12, and IL‐26 [113]. GBM‐PCs also show decreased expression of IL‐2, leading to reduced T cell responses. Further, GBM‐PCs produce TGFβ and hepatocyte growth factor (HGF) to inactivate CD8^+^ T cells [113]. Other PC‐medicated immunosuppressive mechanisms include activation of Tregs and myeloid‐derived suppressive cells (MDSCs) [107, 114]. PCs in TME often exhibit an immature (“synthetic”) phenotype that contributes directly to immunosuppression, immune cell exclusion, and tumor angiogenesis (PMID: 39286984). The discrepancy between healthy mature PCs and tumor‐promoting synthetic PCs reflects the fact that their phenotypic transition is tightly coupled with functional switching. For example, in a mouse melanoma model, phenotypic switching of PCs from a synthetic to a differentiated state improves immune suppression and sensitizes tumors to adoptive T cell therapy, leading to regression of melanoma [90]. PCs induce an unresponsiveness state (anergy) of T cells, which is mediated in part through upregulation of inhibitory checkpoints, specifically the expression of programmed death‐ligand 1 (PD‐L1) and PD‐L2 [115]. In an in vitro coculture system, PCs profoundly inhibit the activation, proliferation, and inflammatory cytokine production, such as IFN‐γ and TNFα  [116]. In tumors, PCs can manifest a strongly immunosuppressive phenotype to support tumor growth and metastasis by allowing tumor cells escaping from immunosurveillance [117]. The crosstalk between PCs and other immune cells, including natural killer (NK) cells, neutrophils, and B lymphocytes are described elsewhere in great details [107]. Together, PCs in tumors navigate broad immunoregulatory networks by interacting with various inflammatory and immune cells.

***Phenotypic switch of pericytes in pancreatic ductal adenocarcinoma***: In the islets of Langerhans, PCs are highly abundant and regulate blood flow, islet cell function, and the extracellular matrix (ECM). These PCs exhibit contractile features by covering nearly 40% of the islet capillaries, and they control blood perfusion through the sympathetic system [14, 118]. However, the PC content is significantly lower in exocrine pancreas compared to endocrine islets of Langerhans. PDAC contains an exceptionally high fibrotic stroma, often constituting 80%–90% of the entire tumor mass [119]. Fibroblasts and ECM create a physical barrier surrounding cancer cells, termed “fibrotic fortress”, which is correlated with anticancer drug resistance, metastasis, and poor prognosis [119]. As mentioned above, the KRAS mutation‐FOXC2‐Ang2 signaling axis ablates PCs in tumors, and over 90% PDAC cases carry KRAS mutations. PDACs generally lack detectable PC‐EC interactions [92, 120]. KRAS mutations in PDACs are considered as an early oncogenic switch to drive tumor growth, metastasis, and drug resistance [121]. A large population of PCs in PDACs exhibits αSMA positivity, which usually absences in microvascular PCs [120]. The loss of the PC‐EC interaction and gain of a αSMA^+^ subpopulation in PDACs indicate phenotypic and functional transitions of regular PCs to myo‐PCs. Interestingly, a study shows that PADCs in human patients with more mature microvessels have improved clinical outcomes, which are consistent with the notion of phenotypic switching of PCs to aberrant PCs [122].

Another subpopulation of PCs i.e., the PeSCs, possess PC, stemness, and stromal cell features, which is barely detectable under physiological conditions, but become abundant in PDAC tissues [123]. PeSCs reduce tumor infiltration of CD4^+^ and CD8^+^ T cells by differentiation into Ly6G^+^ MDSCs, resulting in anti‐PD1 immunotherapy resistance. Notably, PeSCs directly stimulate PDAC‐cancer cell proliferation [123].

### 1.4 Transfer Cascade of Pericytes in Cancer Metastasis

***Pericyte loss in metastatic cell intravasation***: Intravasation of cancer cells through the vessel wall takes place in a sequential event of BM degradation, PC loss, and opening of the inter‐EC junctions (Figure 2). In a genetic mouse model that carries thymidine kinase under the NG2 promoter (NG2‐tk mice), treatment of NG2‐tk mice with ganciclovir selectively ablates NG2^+^ PCs [124]. In this mouse model, PC depletion promotes breast cancer metastatic intravasation by increasing tumor hypoxia, EMT, and c‐Met activation [124]. Other studies support the pro‐metastatic function by perturbing the PC‐EC interaction [26, 125, 126, 127]. Likewise, pharmacological ablation of PCs by imatinib and sunitinib that target the PDGFR‐β signaling also promotes cancer metastasis [124]. Consistent with preclinical findings, clinical studies show a reverse correlation between PC contents and patient survival [42, 128, 129]. Another study reports that endosialin (CD284)‐expressing PCs promote metastatic intravasation without affecting primary tumor growth through a cell‐cell contact‐dependent mechanism [127].

***Pericyte loss in metastatic cell extravasation***: In most organs, extravasation of circulating cells through the microvascular wall occurs by opening the inter‐EC junctions, which are tightly linked by inter‐cellular adhesion molecules [130, 131, 132]. This same mechanism is also utilized by cancer cells for intravasation and extravasation, which are critical steps of the metastatic cascade [130, 132, 133]. Typically, metastatic cancer cells must traverse three barriers, including the endothelial layer, perivascular layer, and BM, in the vessel wall for intravasation or extravasation [134](Figure 2). While intertwined interactions between cancer cells and ECs for cancer invasion receive enormous attention and are relatively well‐studied, the role of PCs in facilitating metastasis remains largely unknown.

A reverse sequence occurs when circulating tumor cells (CTCs) extravasate through the vessel wall in a distal metastatic organ [133]. These opposing sequential processes of intravasation and extravasation may require the cell surface machineries for recognition between cancer cells and vessel wall components. At the time of this writing, little is known about the identities of molecular machineries and their underlying mechanisms in cancer cell intravasation and extravasation, the two critical processes of the metastatic cascade.

***Stimulation of the formation of circulating cancer cell‐neutrophil clusters by PCs***: A single cell‐RNA sequencing approach has identified a subpopulation of nicotinamide N‐methyltransferase (NNMT)^+^ PCs in both human and mouse colorectal cancers (CRCs), which is correlated with high metastatic potentials [8]. Interestingly, human CRC patients with high numbers of NNMT^+^ PC‐blood vessels show intravascular circulating cancer cell (CTC)‐neutrophil clusters, which are poor prognostic markers for CRC patients. Nnmt‐specific deletion in PC inhibits the formation of CTC‐neutrophil clusters and cancer metastasis [8]. Tumor NNMT^+^ PCs also promote transendothelial migration of cancer cells. In‐depth mechanistic study demonstrates that NNMT^+^ PC‐derived CXCL5 mediates the formation of CTC‐neutrophil clusters. Conversely, inhibition of the CXCL5/chemokine‐(C‐X‐C motif) receptor 2 (CXCR2) signaling pathway ablates the CTC‐neutrophil cluster formation and metastasis. Since the CTC‐neutrophil clusters exhibit improved survivals in the circulation and metastatic potentials, the discovery of PC‐modulated CTC‐neutrophil cluster formation provides an example of PCs in the modulation of CTC survivals in the circulation.

**FIGURE 4:**
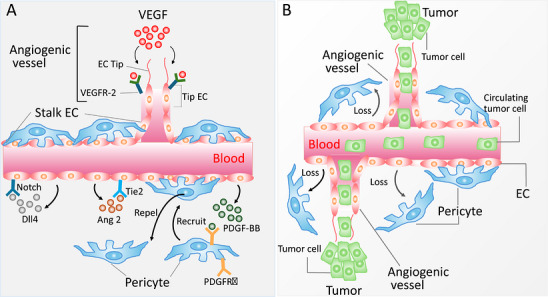
Tumor angiogenesis, pericyte recruitment, and pericyte loss. (A) The VEGF‐VEGFR2‐induced sprouting of angiogenic vessels through the formation of tip endothelial cells. To prevent excessive sprouting, the stalk endothelial cells in the vessel wall are usually stabilized by the PDGFB‐PDGFRβ‐recruited pericytes. The Dll4‐NOTCH1 signaling further prevents excessive sprouting of angiogenic vessels. In contrast to the PDGFB‐PDGFRβ pathway, the Ang2‐Tie2 signaling repels pericyte from blood vessels. (B) Angiogenic vessels are typically disorganized and leaky, which permit tumor cells for intravasation. Pericyte loss further facilitates metastatic cancer cell intravasation. EC = endothelial cell; VEGF = vascular endothelial growth factor; PDGF = platelet‐derived growth factor; Ang 2 = angiopoietin 2.

***PC‐mediated angiogenesis in metastasis***: Under physiological conditions, the PC coverage in microvessels prevents excessive angiogenesis by limiting endothelial sprouting [7, 25, 84](Figure 4). PC coverage in microvessels is tightly regulated by distinctive signaling molecules. For example, angiogenic ECs produce high levels of PDGF‐BB to recruit PCs onto the newly formed microvessels [1, 24] (Figure 4). PC recruitment prevents excessive and undirected vascular sprouting and subsequently angiogenesis. The formation of angiogenic vessels often occurs through a mechanism of angiogenic signaling gradients that guide EC migration toward the cellular source of angiogenic factor gradients [99].

Newly formed angiogenic vessels are highly vulnerable to cancer cell intravasation and eventually metastasis through several possible mechanisms (Figure 4), including (1) lack of sufficient PC coverage; (2) highly leaky; (3) lack of complete BM protection; and (4) feeble inter‐EC junctions. In support of this view, several preclinical studies show that PC‐augmented angiogenesis significantly contributes to cancer metastasis [135, 136, 137, 138].

**FIGURE 5:**
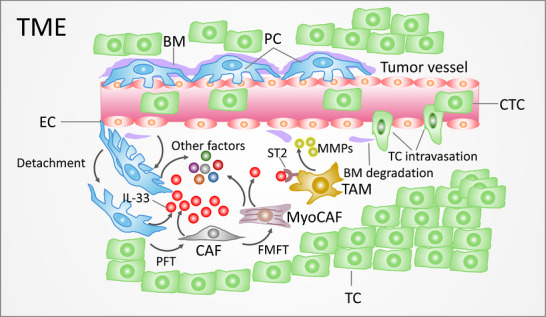
Pericyte‐derived paracrine signaling molecules and phenotypical cell switching in cancer metastasis. In the tumor microenvironment, activated pericytes produce inflammatory cytokines such as interleukin‐33 that recruits inflammatory cells to tumors. IL‐33 also promotes cancer metastasis by inducing the M‐to‐M2 macrophage transition. The IL‐33‐activated macrophages produce high levels of metalloproteinases such as MMP‐9 to degrade the basement membrane. Degradation of the basement membrane further warrants metastatic tumor cell intravasation into the circulation. After detaching from microvessels, pericytes often undergo phenotypic changes and differentiation into other cell types, such as fibroblasts. The pericyte‐to‐fibroblast transition promotes cancer metastasis through mechanisms of the fibroblast‐myofibroblast transition and augmenting tumor inflammation. EC = endothelial cell; PC = pericyte; TC = tumor cell; BM = basement membrane; CTC = circulating tumor cell; IL‐33 = interleukin‐33; PFT = pericyte‐to‐fibroblast transition; CAF = cancer‐associated fibroblast; TAM = tumor‐associated macrophage; MMP = matrix metalloproteinase; FMFT = fibroblast‐to‐myofibroblast transition; TME = tumor microenvironment.

***Pericyte‐TAM interaction in cancer metastasis***: In TME, PCs produce a myriad of growth factors, cytokines, and other signaling molecules to communicate with other surrounding cells, including ECs, cancer cells, cancer‐associated fibroblasts (CAFs), and TAMs to modulate tumor growth, metastasis, and drug responses [57, 108, 139, 140, 141]. In response to various stimuli, expression levels of these signaling molecules can be further elevated. For example, simulation of PCs with PDGF‐BB markedly augments interleukin‐33 (IL‐33) expression [108, 142, 143]. In fact, genome‐wide expression profiling of PDGF‐BB‐stimulated PCs demonstrates that IL‐33 is the most upregulated gene among all genes [108]. Quantitative analysis shows that the expression level of MMP‐9 in the PDGF‐BB‐stimulated PCs increases by over 200 folds [142]. Together with other elevated MMPs, MMP‐9 degrades the BM surrounding microvessels, permitting tumor cell intravasation into the circulation (Figure 5).

TAMs activated by PC‐derived IL‐33 release high levels of CXCL3 that induce the CAF‐to‐myoCAF transition through the CXCR2 receptor [143]. In a mouse PDAC model, CXCL3‐induced myoCAFs promote cancer metastasis through a hijacking‐cancer‐escape mechanism. In addition to IL‐33, PC‐associated other growth factors, cytokines, including chemokines may promote cancer metastasis by altering cellular components in TME. The intimate interactions between PCs and TAMs in TME are further unfolded by a single‐cell RNA sequencing analysis of TAMs derived from a spontaneous mouse mammary tumor, which identified a subset of hyaluronic acid receptor 1 (Lyve‐1)^+^ TAMs [110]. Lyve‐1+ TAMs promote tumor growth by expansion of PDGF‐CC‐dependent perivascular mesenchymal cell‐mediated angiogenesis.

***Pericyte‐mediated cell type switching and matrix remodeling in metastasis***: As mentioned above, PCs with MSC features exhibit high cellular plasticity by differentiation into other cell types, including adipocytes and fibroblasts. It is likely that PCs retain their stemness features in the vessel wall by interacting with ECs [92, 137]. The neuron‐glial antigen (NG2) is one of PC surface markers, which often becomes downregulated after detaching from microvessels [92, 137]. The loss of NG2 positivity after disassociation from tumor microvessels may reflect the fact that NG2^+^PCs differentiate into other NG2^−^ cell types. Genetic tracing of NG2^+^PC demonstrates that PCs in TME differentiate into CAFs and myoCAFs to promote cancer metastasis [9]. This phenotypic change is defined as the pericyte‐to‐fibroblast transition (PFT) [9]. Perhaps, PFT exists in various physiological and pathological processes and contributes to the onset, development, and progression of various diseases.

The existence of heterogeneous tumor PC subpopulations in clinical colorectal cancer samples and other cancers has been uncovered using an integrated approach of single‐cell RNA sequencing (scRNAseq) [90, 144, 145, 146, 147]. Interestingly, a high TCF21 (a family member of the basic helix–loop–helix transcription factors)‐expressing PC subpopulation promotes cancer metastasis by remodeling extracellular matrix and BM [145]. Additionally, PCs facilitate the formation of circulating tumor cell (CTC)‐neutrophil clusters for metastasis [8]. Other studies show that cancer stem cell (CSC)‐derived PCs promote brain metastasis of lung cancers by enhancing trans‐EC migration [148, 149]. Together, multifarious mechanisms are involved in PC‐mediated cancer metastasis, and targeting each of these signaling pathways would potentially provide new opportunities of anti‐metastatic drug development.

**FIGURE 6:**
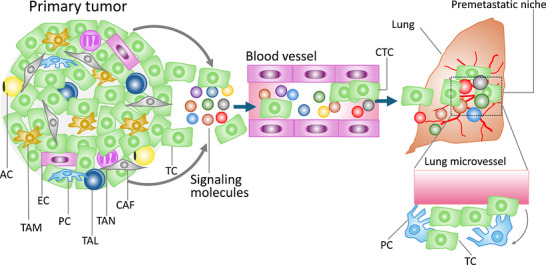
Pericytes in the premetastatic niche formation. In the primary tumor microenvironment, tumor cells and stromal cells produce signaling molecules that enter the circulation. These circulating factors in distant organs manipulate pericytes and other cellular and molecular components, which form a particular microenvironment that allows for the formation of metastatic niches. The activated pericytes in distant organs produce matrix proteins and signaling molecules and to prepare for the arrival of metastatic tumor cells. AC = adipocyte; TAM = tumor‐associated macrophage; CAF = cancer‐associated fibroblast; TC = tumor cell; TAN = tumor‐associated neutrophil; EC = endothelial cell; PC = pericyte; TAL = tumor‐associated lymphocyte; CTC = circulating tumor cell.

***Pericytes in premetastatic niche formation***: Successful colonization of metastatic cancer cells in distal organs is determined by a specific tissue microenvironment that supports tumor cell survival and growth [150, 151, 152, 153]. It is believed that prior to metastasis primary tumors produce signaling molecules to communicate with distal organs to prepare a specific microenvironment, i.e., premetastatic niches, which support metastatic cancer cell colonization and growth [154, 155, 156] (Figure 6). In preclinical metastatic melanoma and metastatic rhabdomyosarcoma models, genetic specific lineage‐tracing shows PCs migrate away from vessels, decrease expression of specific markers and gain proliferation and migration abilities in distant lung tissues [55, 157, 158]. Moreover, the phenotypically activated PCs in distant lung tissues produce high levels of ECM proteins, including fibronectin [125, 158]. Finally, tumor‐derived factors upregulate the expression levels of the Kruppel‐like factor 4 (KLF4), a transcription factor in PCs, which is responsible to preparation of premetastatic niches [157]. It should be emphasized that, in addition to resident cells in distal organs, recruitment of other cells, including various myeloid cells, also actively participates in the formation of premetastatic niches [155, 159, 160, 161]. Additionally, tumor‐derived exosomes also engage in the preparation of premetastatic niches in distant organs [159, 161, 162, 163]. Together with other cell types, PC‐derived factors may also actively take part in the recruitment of myeloid cells for the premetastatic niche formation. PCs in various tissues and organs constitute heterogenous subpopulations, little is known about the roles of various subpopulations in premetastatic niche formation.

### 1.5 Perspectives

Emerging evidence shows that PCs play critical and dynamic roles in the regulation of tumor invasion and metastasis through complex mechanisms of vascular remodeling, pro‐inflammation, phenotypical and functional switching of cell types, and modulation of ECM. PCs are engaged in each process of the metastatic cascade, including intravasation, extravasation, and the premetastatic niche formation. When PCs are associated with tumor microvessels, these perivascular cells upsurge the vessel wall stability and protect microvessels from intravasation of other cells, including malignant cells. However, when PCs migrate away from tumor vessels, they become pro‐metastatic cells by reprogramming the cellular and molecular components of TME. In the primary tumor site, the perivascular location of PCs permits their direct intertwined crosstalk with tumor cells through juxtacrine and paracrine signaling pathways. It seems that PCs are highly responsive to various tumor cell‐derived signaling factors and tumor‐manipulated PCs succor metastasis by coordinating intravasation, extravasation, metastatic niche formation, and probably metastatic regrowth.

While most research efforts focus on understanding the role of PCs in manipulating tumor cell invasion and intravasation, little is known about their functions in metastatic extravasation. Intravasation and extravasation processes mandate the out‐in and in‐out transmigration of cancer cells through the vessel wall. During the in‐out transmigration of CTCs across the microvessel wall, PCs less likely have a direct contact with tumor cells because of the endothelium barrier. At the time of this writing, it is unknown if PCs located in distal organs produce signaling molecules that instruct tumor cells across the vessel wall. After the metastatic niche formation in a distant organ, metastatic nodules need to regrow into a clinically detectable mass. As PCs participate in tumor angiogenesis, inflammation, and fibrosis, it is predicted that these cells significantly contribute to the regrowth of metastases.

Therapeutic targeting PCs for anticancer drug development may not be a straightforward approach due to their high plasticity of cellular and molecular signatures. Owing to the lack of PC‐specific signaling pathways, specific targeting PCs for drug development would be challenging. If drugs targeting PCs are eventually developed, these potential drugs would indistinguishably target PCs located in tumors and healthy tissues. However, defining tumor‐specific PC subpopulations that are responsible for cancer metastasis would provide an exciting opportunity for anticancer drug development. In support of this view, several preclinical studies have defined specific PC subpopulations, including NKX2‐3^high^ tumor PCs, NNMT‐expressing PCs, desmin^+^ PCs, and CD13^+^ PCs, which are involved in various processes of cancer metastasis [8, 125, 145, 164]. It is unclear if pro‐metastatic PC subpopulations exist in all tumors or are limited to a particular cancer type. Thus, targeting pro‐metastatic PC subpopulations as a general approach of anticancer drug development remains conceptually and technically challenging.

PCs in TME are highly plastic and often commit to differentiation into other cell types. Perhaps, PCs often experience the phenotypic switching between the metastasis‐inhibitory and pro‐metastatic PCs. If so, mechanistic understanding and pharmacological targeting of the PC phenotypic switching would provide important clues for the treatment of the metastatic disease. Discovery of molecular machineries and the identity of signaling molecules would provide important information for drug development.

As discussed in the main text, cancer metastasis often occurs when primary tumors are in tiny colonies. For example, visualization of the metastatic cascade at various stages in zebrafish cancer metastasis models shows that intravasation happens when primary tumors are tiny [165, 166, 167, 168]. Reconstitution of the TME with the stromal components of various cells indicates that CTCs often form clusters with other stromal cells, such as TAMs and CAFs [165, 166]. Similarly, the formation of CTC‐neutrophil clusters modulated by PCs provides another example. The early initiation of the metastatic cascade raises a front‐line challenge of targeting PC for the treatment of metastatic diseases. The key question is whether it is too late to target PCs for therapeutic intervention of metastasis. At the time of cancer diagnosis, metastasis may have already occurred and even completed the entire metastatic process, including various stages of the metastatic cascade. If so, would targeting PCs only inhibit new metastasis? At the time of this writing, this important issue remains unanswered.

As PCs display multiple biological functions, including the regulation of angiogenesis, vascular permeability, and immune responses, simultaneous targeting PCs and other cellular components in TME would in theory, improve the therapeutic effects of existing anticancer modalities. In a combination therapeutic setting, agents that neutralize of PC‐mediated immune suppression would improve the outcome of anticancer immunotherapy. Importantly, targeting PCs may even convert immunotherapy‐resistant tumors to sensitive tumors by improving the immunosuppressive microenvironment in various tumors. Similarly, combining antiangiogenic drugs with PC‐targeted agents would also improve therapeutic efficacy for anticancer therapy. In fact, in mouse tumor models anti‐VEGF and anti‐PDGF combination therapy effectively treats anti‐VEGF or anti‐PDGF resistant tumors by simultaneously targeting ECs and PCs [137].

Another important issue of targeting PCs for anticancer therapy is related to vascular permeability. While the protection of tumor vasculatures by PCs against leakage would potentially inhibit metastasis, targeting PCs by drugs would increase vascular permeability. Assuming the circulation is loaded with anticancer drugs, targeting PCs would increase drug extravasation in tumor tissues and thus boost anticancer effects. However, this approach would also increase drug delivery in non‐tumor tissues and possibly increase the risk of cancer metastasis. Nevertheless, during the last few decades, momentous research progress has been made in gaining mechanistic insights into the PC‐mediated cancer metastasis.

## Conflicts of Interest

The authors declare no conflicts of interest.

## Data Availability

The data that support this article are available upon request.

## References

[1] A. Armulik , G. Genove , and C. Betsholtz , “Pericytes: Developmental, Physiological, and Pathological Perspectives, Problems, and Promises,” Developmental Cell 21, no. 2 (2011): 193–215, 10.1016/j.devcel.2011.07.001. 21839917

[2] J. Folkman , C. C. Haudenschild , and B. R. Zetter , “Long‐Term Culture of Capillary Endothelial Cells,” Proceedings of the National Academy of Sciences 76, no. 10 (1979): 5217–5221, 10.1073/pnas.76.10.5217. PMC413111 291937

[3] D. E. Sims , “The Pericyte—A Review,” Tissue and Cell 18, no. 2 (1986): 153–174, 10.1016/0040-8166(86)90026-1. 3085281

[4] E. Trimm and K. Red‐Horse , “Vascular Endothelial Cell Development and Diversity,” Nature Reviews Cardiology 20, no. 3 (2023): 197–210, 10.1038/s41569-022-00770-1. 36198871 PMC9533272

[5] H. G. Augustin and G. Y. Koh , “A Systems View of the Vascular Endothelium in Health and Disease,” Cell 187, no. 18 (2024): 4833–4858, 10.1016/j.cell.2024.07.012. 39241746

[6] K. E. de Visser and J. A. Joyce , “The Evolving Tumor Microenvironment: from Cancer Initiation to Metastatic Outgrowth,” Cancer Cell 41, no. 3 (2023): 374–403, 10.1016/j.ccell.2023.02.016. 36917948

[7] A. Raza , M. J. Franklin , and A. Z. Dudek , “Pericytes and Vessel Maturation during Tumor Angiogenesis and Metastasis,” American Journal of Hematology 85, no. 8 (2010): 593–598, 10.1002/ajh.21745. 20540157

[8] S. Wang , G. Ye , X. Xu , et al., “Pericyte Drives the Formation of Circulating Tumour Cell‐Neutrophil Clusters to Promote Colorectal Cancer Metastasis,” Gut 75, no. 1 (2025): 81–93, 10.1136/gutjnl-2024-334618. 40701795 PMC12703295

[9] K. Hosaka , Y. Yang , T. Seki , et al., “Pericyte–Fibroblast Transition Promotes Tumor Growth and Metastasis,” Proceedings of the National Academy of Sciences 113, no. 38 (2016), 10.1073/pnas.1608384113. PMC5035870 27608497

[10] G. Bergers and S. Song , “The Role of Pericytes in Blood‐Vessel Formation and Maintenance,” Neuro‐Oncology 7, no. 4 (2005): 452–464, 10.1215/S1152851705000232. 16212810 PMC1871727

[11] J. R. Henderson and M. C. Moss , “A Morphometric Study of the Endocrine and Exocrine Capillaries of the Pancreas,” Quarterly Journal of Experimental Physiology 70, no. 3 (1985): 347–356, 10.1113/expphysiol.1985.sp002920. 3898188

[12] M. Schaeffer , D. J. Hodson , C. Lafont , and P. Mollard , “Endocrine Cells and Blood Vessels Work in Tandem to Generate Hormone Pulses,” Journal of Molecular Endocrinology 47, no. 2 (2011): R59–R66, 10.1530/JME-11-0035. 21622530

[13] Y. Cao , “VEGF‐Targeted Cancer Therapeutics—Paradoxical Effects in Endocrine Organs,” Nature Reviews Endocrinology 10, no. 9 (2014): 530–539, 10.1038/nrendo.2014.114. 25048037

[14] J. Almaca , J. Weitz , R. Rodriguez‐Diaz , E. Pereira , and A. Caicedo , “The Pericyte of the Pancreatic Islet Regulates Capillary Diameter and Local Blood Flow,” Cell Metabolism 27, no. 3 (2018): 630–644.e4, 10.1016/j.cmet.2018.02.016. 29514070 PMC5876933

[15] W. C. Aird , “Endothelial Cell Heterogeneity,” Cold Spring Harbor Perspectives in Medicine 2 (2012): a006429, 10.1101/cshperspect.a006429. 22315715 PMC3253027

[16] P. H. Dias Moura Prazeres , I. F. G. Sena , I. D. T. Borges , et al., “Pericytes Are Heterogeneous in Their Origin Within the Same Tissue,” Developmental Biology 427, no. 1 (2017): 6–11, 10.1016/j.ydbio.2017.05.001. 28479340 PMC6076854

[17] G. Collett , A. Wood , M. Y. Alexander , et al., “Receptor Tyrosine Kinase Axl Modulates the Osteogenic Differentiation of Pericytes,” Circulation Research 92, no. 10 (2003): 1123–1129, 10.1161/01.RES.0000074881.56564.46. 12730092

[18] A. Dellavalle , M. Sampaolesi , R. Tonlorenzi , et al., “Pericytes of Human Skeletal Muscle Are Myogenic Precursors Distinct From Satellite Cells,” Nature Cell Biology 9, no. 3 (2007): 255–267, 10.1038/ncb1542. 17293855

[19] C. Farrington‐Rock , N. J. Crofts , M. J. Doherty , B. A. Ashton , C. Griffin‐Jones , and A. E. Canfield , “Chondrogenic and Adipogenic Potential of Microvascular Pericytes,” Circulation 110, no. 15 (2004): 2226–2232, 10.1161/01.CIR.0000144457.55518.E5. 15466630

[20] A. Bouacida , P. Rosset , V. Trichet , et al., “Pericyte‐Like Progenitors Show High Immaturity and Engraftment Potential as Compared with Mesenchymal Stem Cells,” PLoS ONE 7, no. 11 (2012): 48648, 10.1371/journal.pone.0048648. PMC3492496 23144918

[21] M. Herrmann , J. J. Bara , C. M. Sprecher , et al., “Pericyte Plasticity‐Comparative Investigation of the Angiogenic and Multilineage Potential of Pericytes From Different Human Tissues,” European Cells & Materials 31 (2016): 236–249, 10.22203/ecm.v031a16. 27062725

[22] J. Huang , J. O. Bae , J. P. Tsai , et al., “Angiopoietin‐1/Tie‐2 Activation Contributes to Vascular Survival and Tumor Growth During VEGF Blockade,” International Journal of Oncology 34, no. 1 (2009): 79. 19082480 PMC3160826

[23] A. Prete , A. S. Lo , P. M. Sadow , et al., “Pericytes Elicit Resistance to Vemurafenib and Sorafenib Therapy in Thyroid Carcinoma via the TSP‐1/TGFβ1 Axis,” Clinical Cancer Research 24, no. 23 (2018): 6078–6097, 10.1158/1078-0432.CCR-18-0693. 30076136 PMC6279578

[24] A. Armulik , A. Abramsson , and C. Betsholtz , “Endothelial/Pericyte Interactions,” Circulation Research 97, no. 6 (2005): 512–523, 10.1161/01.RES.0000182903.16652.d7. 16166562

[25] H. Gerhardt and C. Betsholtz , “Endothelial‐Pericyte Interactions in Angiogenesis,” Cell and Tissue Research 314, no. 1 (2003): 15–23, 10.1007/s00441-003-0745-x. 12883993

[26] S. Chatterjee and U. P. Naik , “Pericyte‐Endothelial Cell Interaction,” Cell Adhesion & Migration 6, no. 3 (2012): 157–159, 10.4161/cam.20252. 22568989 PMC3427227

[27] A. M. Schor , A. E. Canfield , A. B. Sutton , E. Arciniegas , and T. D. Allen , “Pericyte Differentiation,” Clinical Orthopaedics and Related Research, no. 313 (1995): 81–91. 7543836

[28] A. Geevarghese and I. M. Herman , “Pericyte‐Endothelial Crosstalk: Implications and Opportunities for Advanced Cellular Therapies,” Translational Research 163, no. 4 (2014): 296–306, 10.1016/j.trsl.2014.01.011. 24530608 PMC3976718

[29] K. Hosaka , Y. Yang , T. Seki , et al., “Tumour PDGF‐BB Expression Levels Determine Dual Effects of Anti‐PDGF Drugs on Vascular Remodelling and Metastasis,” Nature Communications 4 (2013): 2129, 10.1038/ncomms3129. 23831851

[30] D. Ferland‐McCollough , S. Slater , J. Richard , C. Reni , and G. Mangialardi , “Pericytes, an Overlooked Player in Vascular Pathobiology,” Pharmacology & Therapeutics 171 (2017): 30–42, 10.1016/j.pharmthera.2016.11.008. 27916653 PMC6008604

[31] S. L. Lin , T. Kisseleva , D. A. Brenner , and J. S. Duffield , “Pericytes and Perivascular Fibroblasts Are the Primary Source of Collagen‐Producing Cells in Obstructive Fibrosis of the Kidney,” The American Journal of Pathology 173, no. 6 (2008): 1617–1627, 10.2353/ajpath.2008.080433. 19008372 PMC2626374

[32] V. S. LeBleu and E. G. Neilson , “Origin and Functional Heterogeneity of Fibroblasts,” The FASEB Journal 34, no. 3 (2020): 3519–3536, 10.1096/fj.201903188R. 32037627

[33] K. Foster , J. Sheridan , H. Veiga‐Fernandes , et al., “Contribution of Neural Crest‐Derived Cells in the Embryonic and Adult Thymus,” The Journal of Immunology 180, no. 5 (2008): 3183–3189, 10.4049/jimmunol.180.5.3183. 18292542

[34] S. M. Muller , C. C. Stolt , G. Terszowski , et al., “Neural Crest Origin of Perivascular Mesenchyme in the Adult Thymus,” The Journal of Immunology 180, no. 8 (2008): 5344–5351, 10.4049/jimmunol.180.8.5344. 18390716

[35] J. Cai , O. Kehoe , G. M. Smith , P. Hykin , and M. E. Boulton , “The Angiopoietin/Tie‐2 System Regulates Pericyte Survival and Recruitment in Diabetic Retinopathy,” Investigative Opthalmology & Visual Science 49, no. 5 (2008): 2163, 10.1167/iovs.07-1206. 18436850

[36] K. S. Volz , A. H. Jacobs , H. I. Chen , et al., “Pericytes Are Progenitors for Coronary Artery Smooth Muscle,” Elife 4 (2015): 10036, 10.7554/eLife.10036. PMC4728130 26479710

[37] Y. Yao , “Challenges in Pericyte Research: Pericyte‐Specific and Subtype‐Specific Markers,” Translational Stroke Research 13, no. 6 (2022): 863–865, 10.1007/s12975-022-01018-3. 35384635 PMC10041340

[38] R. Kramann , R. K. Schneider , D. P. DiRocco , et al., “Perivascular Gli1^+^ Progenitors Are Key Contributors to Injury‐Induced Organ Fibrosis,” Cell Stem Cell 16, no. 1 (2015): 51–66, 10.1016/j.stem.2014.11.004. 25465115 PMC4289444

[39] R. Kramann , J. Wongboonsin , M. Chang‐Panesso , F. G. Machado , and B. D. Humphreys , “Gli1^+^ Pericyte Loss Induces Capillary Rarefaction and Proximal Tubular Injury,” Journal of the American Society of Nephrology 28, no. 3 (2017): 776–784, 10.1681/ASN.2016030297. 27624490 PMC5328159

[40] C. Kuppe , M. M. Ibrahim , J. Kranz , et al., “Decoding Myofibroblast Origins in human Kidney Fibrosis,” Nature 589, no. 7841 (2021): 281–286, 10.1038/s41586-020-2941-1. 33176333 PMC7611626

[41] N. Guimaraes‐Camboa , P. Cattaneo , Y. Sun , et al., “Pericytes of Multiple Organs Do Not Behave as Mesenchymal Stem Cells in Vivo,” Cell Stem Cell 20, no. 3 (2017): 345–359.e5, 10.1016/j.stem.2016.12.006. 28111199 PMC5337131

[42] Y. Yonenaga , A. Mori , H. Onodera , et al., “Absence of Smooth Muscle Actin‐Positive Pericyte Coverage of Tumor Vessels Correlates with Hematogenous Metastasis and Prognosis of Colorectal Cancer Patients,” Oncology 69, no. 2 (2005): 159–166, 10.1159/000087840. 16127287

[43] Z. Shi , S. Xiong , R. Hu , et al., “The Notch‐PDGFRβ Axis Suppresses Brown Adipocyte Progenitor Differentiation in Early Post‐Natal Mice,” Developmental Cell 59, no. 10 (2024): 1233–1251.e5, 10.1016/j.devcel.2024.03.012. 38569546 PMC11874136

[44] H. C. Etchevers , "Pericyte Ontogeny: The Use of Chimeras to Track a Cell Lineage of Diverse Germ Line Origins," In Pericytes: Methods and Protocols (Springer, 2021): 61, 10.1007/978-1-0716-1056-5_6. 33576971

[45] T. Yamazaki , A. Nalbandian , Y. Uchida , et al., “Tissue Myeloid Progenitors Differentiate into Pericytes Through TGF‐β Signaling in Developing Skin Vasculature,” Cell Reports 18, no. 12 (2017): 2991–3004, 10.1016/j.celrep.2017.02.069. 28329690 PMC5393447

[46] H. M. Eilken , S. Nishikawa , and T. Schroeder , “Continuous Single‐Cell Imaging of Blood Generation from Haemogenic Endothelium,” Nature 457, no. 7231 (2009): 896–900, 10.1038/nature07760. 19212410

[47] A. Birbrair , T. Zhang , Z. M. Wang , et al., “Skeletal Muscle Pericyte Subtypes Differ in Their Differentiation Potential,” Stem Cell Research 10, no. 1 (2013): 67–84, 10.1016/j.scr.2012.09.003. 23128780 PMC3781014

[48] A. Birbrair , T. Zhang , Z. M. Wang , M. L. Messi , A. Mintz , and O. Delbono , “Pericytes: Multitasking Cells in the Regeneration of Injured, Diseased, and Aged Skeletal Muscle,” Frontiers in Aging Neuroscience 6 (2014): 245, 10.3389/fnagi.2014.00245. 25278877 PMC4166895

[49] A. Birbrair , T. Zhang , Z.‐M. Wang , M. L. Messi , A. Mintz , and O. Delbono , “Pericytes at the Intersection Between Tissue Regeneration and Pathology,” Clinical Science 128, no. 2 (2015) 81–93, 10.1042/CS20140278. 25236972 PMC4200531

[50] A. Birbrair , “Pericyte Biology: Development, Homeostasis, and Disease,” Advances in Experimental Medicine and Biology 1109 (2018): 1–3, 10.1007/978-3-030-02601-1_1. 30523585

[51] G. Mangialardi , A. Cordaro , and P. Madeddu , “The Bone Marrow Pericyte: An Orchestrator of Vascular Niche,” Regenerative Medicine 11, no. 8 (2016): 883–895, 10.2217/rme-2016-0121. 27885901 PMC5677781

[52] A. M. Jordan , R. J. G. Drake , and K. M. Hodivala‐Dilke , “Angiocrine and Pericrine Signaling: How Endothelial Cells and Pericytes Drive Cancer Progression and Therapy Resistance,” Physiological Reviews 106, no. 1 (2026): 87–119, 10.1152/physrev.00046.2024. 40952787

[53] R. J. Bodnar , L. Satish , C. C. Yates , and A. Wells , “Pericytes: A Newly Recognized Player in Wound Healing,” Wound Repair and Regeneration 24, no. 2 (2016): 204–214, 10.1111/wrr.12415. 26969517 PMC5036393

[54] Y. Tang , C. Frisendahl , T. T. Piltonen , R. K. Arffman , P. G. Lalitkumar , and K. Gemzell‐Danielsson , Cells 13, no. 17 (2024): 1510, 10.3390/cells13171510. 39273080 PMC11394273

[55] Z. Jiang , J. Zhou , L. Li , et al., “Pericytes in the Tumor Microenvironment,” Cancer Letters 556 (2023): 216074, 10.1016/j.canlet.2023.216074. 36682706

[56] J. Rustenhoven , D. Jansson , L. C. Smyth , and M. Dragunow , “Brain Pericytes as Mediators of Neuroinflammation,” Trends in Pharmacological Sciences 38, no. 3 (2017): 291–304, 10.1016/j.tips.2016.12.001. 28017362

[57] S. G. Rayner , C. F. Hung , W. C. Liles , and W. A. Altemeier , “Lung Pericytes as Mediators of Inflammation,” American Journal of Physiology‐Lung Cellular and Molecular Physiology 325, no. 1 (2023): L1–L8, 10.1152/ajplung.00354.2022. 37130806 PMC10292969

[58] H. He , W. Yang , N. Su , et al., “Activating NO–sGC Crosstalk in the Mouse Vascular Niche Promotes Vascular Integrity and Mitigates Acute Lung Injury,” Journal of Experimental Medicine 220, no. 2 (2023), 10.1084/jem.20211422. PMC9984546 36350314

[59] N. Ferrara , “Vascular Endothelial Growth Factor: Basic Science and Clinical Progress,” Endocrine Reviews 25, no. 4 (2004): 581–611, 10.1210/er.2003-0027. 15294883

[60] N. Ferrara , H. P. Gerber , and J. LeCouter , “The Biology of VEGF and Its Receptors,” Nature Medicine 9, no. 6 (2003): 669–676, 10.1038/nm0603-669. 12778165

[61] Y. Cao , “Positive and Negative Modulation of Angiogenesis by VEGFR1 Ligands,” Science Signaling 2, no. 59 (2009), 10.1126/scisignal.259re1. 19244214

[62] W. Risau , H. Drexler , V. Mironov , et al., “Platelet‐Derived Growth Factor Is Angiogenic in Vivo,” Growth Factors 7, no. 4 (1992): 261–266, 10.3109/08977199209046408. 1284870

[63] P. Guo , B. Hu , W. Gu , et al., “Platelet‐Derived Growth Factor‐B Enhances Glioma Angiogenesis by Stimulating Vascular Endothelial Growth Factor Expression in Tumor Endothelia and by Promoting Pericyte Recruitment,” The American Journal of Pathology 162, no. 4 (2003): 1083–1093, 10.1016/S0002-9440(10)63905-3. 12651601 PMC1851242

[64] P. Lindahl , B. R. Johansson , P. Leveen , and C. Betsholtz , “Pericyte Loss and Microaneurysm Formation in PDGF‐B‐Deficient Mice,” Science 277, no. 5323 (1997): 242–245, 10.1126/science.277.5323.242. 9211853

[65] R. Cao , E. Brakenhielm , R. Pawliuk , et al., “Angiogenic Synergism, Vascular Stability and Improvement of Hind‐limb Ischemia by a Combination of PDGF‐BB and FGF‐2,” Nature Medicine 9, no. 5 (2003): 604–613, 10.1038/nm848. 12669032

[66] H. P. Hammes , J. Lin , P. Wagner , et al., “Angiopoietin‐2 Causes Pericyte Dropout in the Normal Retina,” Diabetes 53, no. 4 (2004): 1104–1110, 10.2337/diabetes.53.4.1104. 15047628

[67] E. Iivanainen , L. Nelimarkka , V. Elenius , et al., “Angiopoietin‐Regulated Recruitment of Vascular Smooth Muscle Cells by Endothelial‐Derived Heparin Binding EGF‐like Growth Factor,” The FASEB Journal 17, no. 12 (2003): 1609–1621, 10.1096/fj.02-0939com. 12958167

[68] N. Song , Y. Huang , H. Shi , et al., “Overexpression of Platelet‐Derived Growth Factor‐BB Increases Tumor Pericyte Content via Stromal‐Derived Factor‐1α/CXCR4 Axis,” Cancer Research 69, no. 15 (2009): 6057–6064, 10.1158/0008-5472.CAN-08-2007. 19584297

[69] A. N. Stratman , M. J. Davis , and G. E. Davis , “VEGF and FGF Prime Vascular Tube Morphogenesis and Sprouting Directed by Hematopoietic Stem Cell Cytokines,” Blood 117, no. 14 (2011): 3709–3719, 10.1182/blood-2010-11-316752. 21239704 PMC3083293

[70] A. N. Stratman , A. E. Schwindt , K. M. Malotte , and G. E. Davis , “Endothelial‐Derived PDGF‐BB and HB‐EGF Coordinately Regulate Pericyte Recruitment during Vasculogenic Tube Assembly and Stabilization,” Blood 116, no. 22 (2010): 4720–4730, 10.1182/blood-2010-05-286872. 20739660 PMC2996127

[71] I. M. Moya , L. Umans , E. Maas , et al., “Stalk Cell Phenotype Depends on Integration of Notch and Smad1/5 Signaling Cascades,” Developmental Cell 22, no. 3 (2012): 501–514, 10.1016/j.devcel.2012.01.007. 22364862 PMC4544746

[72] J. D. Leslie , L. Ariza‐McNaughton , A. L. Bermange , R. McAdow , S. L. Johnson , and J. Lewis , “Endothelial Signalling by the Notch Ligand Delta‐Like 4 Restricts Angiogenesis,” Development 134 (2007): 839, 10.1242/dev.003244. 17251261

[73] S. Suchting , C. Freitas , F. le Noble , et al., “The Notch Ligand Delta‐Like 4 Negatively Regulates Endothelial Tip Cell Formation and Vessel Branching,” Proceedings of the National Academy of Sciences 104, no. 9 (2007): 3225–3230, 10.1073/pnas.0611177104. PMC1805603 17296941

[74] R. Benedito , C. Roca , I. Sorensen , et al., “The Notch Ligands Dll4 and Jagged1 Have Opposing Effects on Angiogenesis,” Cell 137, no. 6 (2009): 1124–1135, 10.1016/j.cell.2009.03.025. 19524514

[75] C. F. Wu , W. C. Chiang , C. F. Lai , et al., “Transforming Growth Factor β‐1 Stimulates Profibrotic Epithelial Signaling to Activate Pericyte‐Myofibroblast Transition in Obstructive Kidney Fibrosis,” The American Journal of Pathology 182, no. 1 (2013): 118–131, 10.1016/j.ajpath.2012.09.009. 23142380 PMC3538028

[76] S. C. Shih , M. Ju , N. Liu , J. R. Mo , J. J. Ney , and L. E. Smith , “Transforming Growth Factor β1 Induction of Vascular Endothelial Growth Factor Receptor 1: Mechanism of Pericyte‐induced Vascular Survival In Vivo,” Proceedings of the National Academy of Sciences 100, no. 26 (2003): 15859–15864, 10.1073/pnas.2136855100. PMC307658 14657382

[77] M. Kim , B. Allen , E. A. Korhonen , et al., “Opposing Actions of Angiopoietin‐2 on Tie2 Signaling and FOXO1 Activation,” Journal of Clinical Investigation 126, no. 9 (2016): 3511–3525, 10.1172/JCI84871. 27548529 PMC5004955

[78] C. Suri , P. F. Jones , S. Patan , et al., “Requisite Role of Angiopoietin‐1, a Ligand for the TIE2 Receptor, During Embryonic Angiogenesis,” Cell 87, no. 7 (1996): 1171–1180, 10.1016/s0092-8674(00)81813-9. 8980224

[79] P. C. Maisonpierre , C. Suri , P. F. Jones , et al., “Angiopoietin‐2, a Natural Antagonist for Tie2 That Disrupts In Vivo Angiogenesis,” Science 277, no. 5322 (1997): 55–60, 10.1126/science.277.5322.55. 9204896

[80] M. Teichert , L. Milde , A. Holm , et al., “Pericyte‐Expressed Tie2 Controls Angiogenesis and Vessel Maturation,” Nature Communications 8 (2017): 16106, 10.1038/ncomms16106. PMC5520106 28719590

[81] E. M. Meijer , C. G. M. van Dijk , R. Kramann , M. C. Verhaar , and C. Cheng , “Implementation of Pericytes in Vascular Regeneration Strategies,” Tissue Engineering Part B: Reviews 28, no. 1 (2022): 1–21, 10.1089/ten.TEB.2020.0229. 33231500

[82] Y. Wu , J. Fu , Y. Huang , et al., “Biology and Function of Pericytes in the Vascular Microcirculation,” Animal Models and Experimental Medicine 6, no. 4 (2023): 337–345, 10.1002/ame2.12334. 37317664 PMC10486323

[83] Y. Cao , “Mechanistic Understanding of Crosstalk between Blood Vessels and Immunity and Inflammation in Diseases,” Immunity & Inflammation 2, no. 1 (2026): 6, 10.1007/s44466-025-00021-1.

[84] P. C. Stapor , R. S. Sweat , D. C. Dashti , A. M. Betancourt , and W. L. Murfee , “Pericyte Dynamics during Angiogenesis: New Insights from New Identities,” Journal of Vascular Research 51, no. 3 (2014): 163–174, 10.1159/000362276. 24853910 PMC4149862

[85] T. Ishii , S. Yuge , K. Ando , W. Zhou , and S. Fukuhara , “Pericyte‐Mediated Regulation of Angiogenesis During Cutaneous Wound Healing in Adult Zebrafish,” Communications Biology 8, no. 1 (2025): 1101, 10.1038/s42003-025-08517-7. 40715583 PMC12297410

[86] R. Cao , Y. Xue , E. M. Hedlund , et al., “VEGFR1–Mediated Pericyte Ablation Links VEGF and PlGF to Cancer‐Associated Retinopathy,” Proceedings of the National Academy of Sciences 107, no. 2 (2010): 856–861, 10.1073/pnas.0911661107. PMC2818941 20080765

[87] A. Armulik , G. Genove , M. Mae , et al., “Pericytes Regulate the Blood–Brain Barrier,” Nature 468, no. 7323 (2010): 557–561, 10.1038/nature09522. 20944627

[88] Z. Zhao , A. R. Nelson , C. Betsholtz , and B. V. Zlokovic , “Establishment and Dysfunction of the Blood‐Brain Barrier,” Cell 163, no. 5 (2015): 1064–1078, 10.1016/j.cell.2015.10.067. 26590417 PMC4655822

[89] T. A. Longden , G. Zhao , A. Hariharan , and W. J. Lederer , “Pericytes and the Control of Blood Flow in Brain and Heart,” Annual Review of Physiology 85 (2023): 137–164, 10.1146/annurev-physiol-031522-034807. PMC10280497 36763972

[90] Z. J. Li , B. He , A. Domenichini , et al., “Pericyte Phenotype Switching Alleviates Immunosuppression and Sensitizes Vascularized Tumors to Immunotherapy in Preclinical Models,” Journal of Clinical Investigation 134, no. 18 (2024), 10.1172/JCI179860. PMC11405053 39286984

[91] H. Mahaki , S. Nobari , H. Tanzadehpanah , et al., “Targeting VEGF Signaling for Tumor Microenvironment Remodeling and Metastasis Inhibition: Therapeutic Strategies and Insights,” Biomedicine & Pharmacotherapy 186 (2025): 118023, 10.1016/j.biopha.2025.118023. 40164047

[92] K. Hosaka , P. Andersson , J. Wu , et al., “KRAS Mutation‐Driven Angiopoietin 2 Bestows Anti‐VEGF Resistance in Epithelial Carcinomas,” Proceedings of the National Academy of Sciences 120, no. 29 (2023): 2303740120, 10.1073/pnas.2303740120. PMC10629547 37428914

[93] A. Figueras , M. A. Arbos , M. T. Quiles , F. Viñals , J. R. Germà , and G. Capellà , “The Impact of KRAS Mutations on VEGF‐A Production and Tumour Vascular Network,” BMC cancer 13, no. 1 (2013): 125, 10.1186/1471-2407-13-125. 23506169 PMC3610256

[94] A. M. Li , A. Boichard , and R. Kurzrock , “Mutated TP53 Is a Marker of Increased VEGF Expression: Analysis of 7,525 Pan‐Cancer Tissues,” Cancer Biology & Therapy 21, no. 1 (2020): 95–100, 10.1080/15384047.2019.1665956. 31564192 PMC7012180

[95] M. F. Ghahremani , S. Goossens , D. Nittner , et al., “p53 Promotes VEGF Expression and Angiogenesis in the Absence of an Intact p21‐Rb Pathway,” Cell Death & Differentiation 20, no. 7 (2013): 888–897, 10.1038/cdd.2013.12. 23449391 PMC3679451

[96] N. Chalkidi , A. Stavropoulou , V.‐Z. Arvaniti , et al., “Notch3 Regulates Pericyte Phenotypic Plasticity in Colorectal Cancer,” Communications Biology 9, no. 1 (2026): 343, 10.1038/s42003-026-09629-4. 41618002 PMC12960917

[97] C. W. Pugh and P. J. Ratcliffe , “Regulation of Angiogenesis by Hypoxia: Role of the HIF System,” Nature Medicine 9, no. 6 (2003): 677–684, 10.1038/nm0603-677. 12778166

[98] Y. Makino , R. Cao , K. Svensson , et al., “Inhibitory PAS Domain Protein Is a Negative Regulator of Hypoxia‐inducible Gene Expression,” Nature 414, no. 6863 (2001): 550–554, 10.1038/35107085. 11734856

[99] H. Gerhardt , M. Golding , M. Fruttiger , et al., “VEGF Guides Angiogenic Sprouting Utilizing Endothelial Tip Cell Filopodia,” The Journal of Cell Biology 161, no. 6 (2003): 1163–1177, 10.1083/jcb.200302047. 12810700 PMC2172999

[100] Y. S. Song , S. Wang , S. Inampudi , H. Risa , C. M. Sorenson , and N. Sheibani , “Pericyte Expression of VEGF‐A Minimally Impacts Ocular Vascular Development and Neovascularization,” Cells 14, no. 18 (2025): 1473, 10.3390/cells14181473. 41002438 PMC12469127

[101] J. I. Greenberg , D. J. Shields , S. G. Barillas , et al., “A Role for VEGF as a Negative Regulator of Pericyte Function and Vessel Maturation,” Nature 456, no. 7223 (2008): 809–813, 10.1038/nature07424. 18997771 PMC2605188

[102] Q. Shi , X. Le , B. Wang , et al., “Regulation of Vascular Endothelial Growth Factor Expression by Acidosis in Human Cancer Cells,” Oncogene 20, no. 28 (2001): 3751–3756, 10.1038/sj.onc.1204500. 11439338

[103] Y. Cao , “Tumor Angiogenesis and Molecular Targets for Therapy,” Frontiers in Bioscience 14 (2009): 3962–3973, 10.2741/3504. 19273326

[104] T. Lechertier , L. E. Reynolds , H. Kim , et al., “Pericyte FAK Negatively Regulates Gas6/Axl Signalling to Suppress Tumour Angiogenesis and Tumour Growth,” Nature Communications 11, no. 1 (2020): 2810, 10.1038/s41467-020-16618-6. PMC7272651 32499572

[105] P. P. Wong , J. M. Munoz‐Felix , M. Hijazi , et al., “Cancer Burden Is Controlled by Mural Cell‐β3‐Integrin Regulated Crosstalk with Tumor Cells,” Cell 181, no. 6 (2020): 1346–1363.e21, 10.1016/j.cell.2020.02.003. 32473126

[106] A. Abramsson , P. Lindblom , and C. Betsholtz , “Endothelial and Nonendothelial Sources of PDGF‐B Regulate Pericyte Recruitment and Influence Vascular Pattern Formation in Tumors,” Journal of Clinical Investigation 112, no. 8 (2003): 1142–1151, 10.1172/JCI18549. 14561699 PMC213487

[107] M. Moro , F. C. Balestrero , and A. A. Grolla , “Pericytes: Jack‐of‐All‐Trades in Cancer‐Related Inflammation,” Frontiers in Pharmacology 15 (2024): 1426033, 10.3389/fphar.2024.1426033. 39086395 PMC11288921

[108] Y. Yang , P. Andersson , K. Hosaka , et al., “The PDGF‐BB‐SOX7 Axis‐Modulated IL‐33 in Pericytes and Stromal Cells Promotes Metastasis Through Tumour‐Associated Macrophages,” Nature Communications 7 (2016): 11385, 10.1038/ncomms11385. PMC4859070 27150562

[109] J. S. Park , I. K. Kim , S. Han , et al., “Normalization of Tumor Vessels by Tie2 Activation and Ang2 Inhibition Enhances Drug Delivery and Produces a Favorable Tumor Microenvironment,” Cancer Cell 30, no. 6 (2016): 953–967, 10.1016/j.ccell.2016.10.018. 27960088

[110] J. W. Opzoomer , J. E. Anstee , I. Dean , et al., “Macrophages Orchestrate the Expansion of a Proangiogenic Perivascular Niche During Cancer Progression,” Science Advances 7, no. 45 (2021), 10.1126/sciadv.abg9518. PMC8565907 34730997

[111] F. Yotsumoto , W. K. You , P. Cejudo‐Martin , K. Kucharova , K. Sakimura , and W. B. Stallcup , “NG2 Proteoglycan‐Dependent Recruitment of Tumor Macrophages Promotes Pericyte‐Endothelial Cell Interactions Required for Brain Tumor Vascularization,” Oncoimmunology 4 (2015): 1001204, 10.1080/2162402X.2014.1001204. PMC4485789 26137396

[112] C. Zhu , I. Chrifi , D. Mustafa , et al., “CECR1‐Mediated Cross Talk Between Macrophages and Vascular Mural Cells Promotes Neovascularization in Malignant Glioma,” Oncogene 36, no. 38 (2017): 5356–5368, 10.1038/onc.2017.145. 28534507 PMC5611481

[113] R. Valdor , D. Garcia‐Bernal , C. Bueno , et al., “Glioblastoma Progression Is Assisted by Induction of Immunosuppressive Function of Pericytes Through Interaction with Tumor Cells,” Oncotarget 8, no. 40 (2017): 68614–68626, 10.18632/oncotarget.19804. 28978142 PMC5620282

[114] J. Hong , N. P. Tobin , H. Rundqvist , et al., “Role of Tumor Pericytes in the Recruitment of Myeloid‐Derived Suppressor Cells,” Journal of the National Cancer Institute 107, no. 10 (2015): djv209, 10.1093/jnci/djv209. 26296362 PMC6592827

[115] H. Domev , I. Milkov , J. Itskovitz‐Eldor , and A. Dar , “Immunoevasive Pericytes from Human Pluripotent Stem Cells Preferentially Modulate Induction of Allogeneic Regulatory T Cells,” Stem Cells Translational Medicine 3, no. 10 (2014): 1169–1181, 10.5966/sctm.2014-0097. 25205843 PMC4181403

[116] Z. Tu , Y. Li , D. S. Smith , et al., “Retinal Pericytes Inhibit Activated T Cell Proliferation,” Investigative Opthalmology & Visual Science 52, no. 12 (2011): 9005, 10.1167/iovs.11-8008. PMC3231798 22003106

[117] S. A. Dabravolski , E. R. Andreeva , A. M. Markin , A. N. Orekhov , and A. A. Melnichenko , “The Role of Pericytes in Regulation of Innate and Adaptive Immunity,” Biomedicines 11, no. 2 (2023): 600, 10.3390/biomedicines11020600. 36831136 PMC9953719

[118] A. Tamayo , L. M. Goncalves , R. Rodriguez‐Diaz , et al., “Pericyte Control of Blood Flow in Intraocular Islet Grafts Impacts Glucose Homeostasis in Mice,” Diabetes 71, no. 8 (2022): 1679–1693, 10.2337/db21-1104. 35587617 PMC9490358

[119] K. K. Myo Min , C. B. Ffrench , C. F. Jessup , M. Shepherdson , S. G. Barreto , and C. S. Bonder , “Overcoming the Fibrotic Fortress in Pancreatic Ductal Adenocarcinoma: Challenges and Opportunities,” Cancers 15, no. 8 (2023): 2354, 10.3390/cancers15082354. 37190281 PMC10137060

[120] V. Natarajan , S. Ha , A. Delgado , et al., “Acquired αSMA Expression in Pericytes Coincides With Aberrant Vascular Structure and Function in Pancreatic Ductal Adenocarcinoma,” Cancers 14, no. 10 (2022): 2448, 10.3390/cancers14102448. 35626052 PMC9139959

[121] J. Luo , “KRAS Mutation in Pancreatic Cancer,” Seminars in Oncology 48, no. 1 (2021): 10–18, 10.1053/j.seminoncol.2021.02.003. 33676749 PMC8380752

[122] E. Katsuta , Q. Qi , X. Peng , S. N. Hochwald , L. Yan , and K. Takabe , “Pancreatic Adenocarcinomas with Mature Blood Vessels Have Better Overall Survival,” Scientific Reports 9 (2019): 1310, 10.1038/s41598-018-37909-5. 30718678 PMC6362082

[123] Z. Wu , K. Thierry , S. Bachy , et al., “Pericyte Stem Cells Induce Ly6G^+^ Cell Accumulation and Immunotherapy Resistance in Pancreatic Cancer,” EMBO reports 24, no. 4 (2023): 56524, 10.15252/embr.202256524. PMC10074138 36802267

[124] V. G. Cooke , V. S. LeBleu , D. Keskin , et al., “Pericyte Depletion Results in Hypoxia‐Associated Epithelial‐to‐Mesenchymal Transition and Metastasis Mediated by Met Signaling Pathway,” Cancer Cell 21, no. 1 (2012): 66–81, 10.1016/j.ccr.2011.11.024. 22264789 PMC3999522

[125] X. Li , S. Yan , X. Wu , et al., “Pericytes Promote Metastasis by Regulating Tumor Local Vascular Tone and Hemodynamics,” Nature Communications 16, no. 1 (2025): 7115, 10.1038/s41467-025-62475-6. PMC12318036 40753165

[126] X. Xian , J. Hakansson , A. Stahlberg , et al., “Pericytes Limit Tumor Cell Metastasis,” Journal of Clinical Investigation 116, no. 3 (2006): 642–651, 10.1172/JCI25705. 16470244 PMC1361347

[127] C. Viski , C. Konig , M. Kijewska , C. Mogler , C. M. Isacke , and H. G. Augustin , “Endosialin‐Expressing Pericytes Promote Metastatic Dissemination,” Cancer Research 76, no. 18 (2016): 5313–5325, 10.1158/0008-5472.CAN-16-0932. 27635044

[128] M. B. O'Keeffe , A. H. Devlin , A. J. Burns , et al., “Investigation of Pericytes, Hypoxia, and Vascularity in Bladder Tumors: Association with Clinical Outcomes,” Oncology Research Featuring Preclinical and Clinical Cancer Therapeutics 17, no. 3 (2008): 93–101, 10.3727/096504008785055530. 18669161

[129] I. M. Stefansson , H. B. Salvesen , and L. A. Akslen , “Vascular Proliferation Is Important for Clinical Progress of Endometrial Cancer,” Cancer Research 66, no. 6 (2006): 3303–3309, 10.1158/0008-5472.CAN-05-1163. 16540684

[130] S. P. Chiang , R. M. Cabrera , and J. E. Segall , “Tumor Cell Intravasation,” American Journal of Physiology‐Cell Physiology 311, no. 1 (2016): C1–C14, 10.1152/ajpcell.00238.2015. 27076614 PMC4967137

[131] G. Bazzoni and E. Dejana , “Endothelial Cell‐to‐Cell Junctions: Molecular Organization and Role in Vascular Homeostasis,” Physiological Reviews 84, no. 3 (2004): 869–901, 10.1152/physrev.00035.2003. 15269339

[132] E. Dejana , F. Orsenigo , C. Molendini , P. Baluk , and D. M. McDonald , “Organization and Signaling of Endothelial Cell‐to‐Cell Junctions in Various Regions of the Blood and Lymphatic Vascular Trees,” Cell and Tissue Research 335, no. 1 (2009): 17–25, 10.1007/s00441-008-0694-5. 18855014 PMC4422058

[133] A. Dupas , J. G. Goetz , and N. Osmani , “Extravasation of Immune and Tumor Cells From an Endothelial Perspective,” Journal of Cell Science 137, no. 21 (2024), 10.1242/jcs.262066. 39530179

[134] N. Reymond , B. B. d'Agua , and A. J. Ridley , “Crossing the Endothelial Barrier During Metastasis,” Nature Reviews Cancer 13, no. 12 (2013): 858–870, 10.1038/nrc3628. 24263189

[135] G. Bergers , S. Song , N. Meyer‐Morse , E. Bergsland , and D. Hanahan , “Benefits of Targeting Both Pericytes and Endothelial Cells in the Tumor Vasculature with Kinase Inhibitors,” Journal of Clinical Investigation 111, no. 9 (2003): 1287–1295, 10.1172/JCI17929. 12727920 PMC154450

[136] C. Lu , A. A. Kamat , Y. G. Lin , et al., “Dual Targeting of Endothelial Cells and Pericytes in Antivascular Therapy for Ovarian Carcinoma,” Clinical Cancer Research 13, no. 14 (2007): 4209–4217, 10.1158/1078-0432.CCR-07-0197. 17634550

[137] K. Hosaka , Y. Yang , M. Nakamura , et al., “Dual Roles of Endothelial FGF‐2–FGFR1–PDGF‐BB and Perivascular FGF‐2–FGFR2–PDGFRβ Signaling Pathways in Tumor Vascular Remodeling,” Cell Discovery 4 (2018), 10.1038/s41421-017-0002-1. PMC5798893 29423271

[138] K. Hosaka , Y. Yang , T. Seki , et al., “Therapeutic Paradigm of Dual Targeting VEGF and PDGF for Effectively Treating FGF‐2 Off‐Target Tumors,” Nature Communications 11, no. 1 (2020): 3704, 10.1038/s41467-020-17525-6. PMC7382445 32709869

[139] E. Gil , C. Venturini , D. Stirling , et al., “Pericyte Derived Chemokines Amplify Neutrophil Recruitment across the Cerebrovascular Endothelial Barrier,” Frontiers in Immunology 13 (2022): 935798, 10.3389/fimmu.2022.935798. 35967327 PMC9371542

[140] V. Volarevic , C. R. Harrell , A. Arsenijevic , and V. Djonov , “An Interplay between Pericytes, Mesenchymal Stem Cells, and Immune Cells in the Process of Tissue Regeneration,” Analytical Cellular Pathology 2025 (2025): 4845416, 10.1155/ancp/4845416. PMC12003036 40241723

[141] H. van Splunder , P. Villacampa , A. Martinez‐Romero , and M. Graupera , “Pericytes in the Disease Spotlight,” Trends in Cell Biology 34, no. 1 (2024): 58–71, 10.1016/j.tcb.2023.06.001. 37474376 PMC10777571

[142] P. Andersson , Y. Yang , K. Hosaka , et al., “Molecular Mechanisms of IL‐33–Mediated Stromal Interactions in Cancer Metastasis,” JCI Insight 3, no. 20 (2018), 10.1172/jci.insight.122375. PMC6237443 30333314

[143] X. Sun , X. He , Y. Zhang , et al., “Inflammatory Cell‐Derived CXCL3 Promotes Pancreatic Cancer Metastasis Through a Novel Myofibroblast‐Hijacked Cancer Escape Mechanism,” Gut 71, no. 1 (2022): 129–147, 10.1136/gutjnl-2020-322744. 33568427

[144] K. Xiong , B. Pan , H. Fang , and Z. Tao , “Single‐Cell Sequencing Analysis Reveals Cancer‐Associated Pericyte Subgroup in Esophageal Squamous Cell Carcinoma to Predict Prognosis,” Frontiers in Immunology 15 (2024): 1474673, 10.3389/fimmu.2024.1474673. 39835116 PMC11743493

[145] X. Li , J. Pan , T. Liu , et al., “Novel TCF21 High Pericyte Subpopulation Promotes Colorectal Cancer Metastasis by Remodelling Perivascular Matrix,” Gut 72, no. 4 (2023): 710–721, 10.1136/gutjnl-2022-327913. 36805487 PMC10086488

[146] K. Del Toro , Y. Licon‐Munoz , W. Crabtree , T. Oper , C. Robbins , and W. C. Hines , “Breast Pericytes: A Newly Identified Driver of Tumor Cell Proliferation,” Frontiers in oncology 14 (2024): 1455484, 10.3389/fonc.2024.1455484. 39741968 PMC11685225

[147] J. Kim , P. C. de Sampaio , D. M. Lundy , et al., “Heterogeneous Perivascular Cell Coverage Affects Breast Cancer Metastasis and Response to Chemotherapy,” JCI Insight 1, no. 21 (2016): 90733, 10.1172/jci.insight.90733. 28018977 PMC5161212

[148] Q. Huang , L. Liu , D. Xiao , et al., “CD44+ lung Cancer Stem Cell‐Derived Pericyte‐Like Cells Cause Brain Metastases Through GPR124‐Enhanced Trans‐Endothelial Migration,” Cancer Cell 41, no. 9 (2023): 1621–1636.e8, 10.1016/j.ccell.2023.07.012. 37595587

[149] W. Liu , W. Duan , S. Xia , et al., “CD146 + Pericyte‐Like Lung Cancer Brain Metastatic Stem Cells Promote Tumor Angiogenesis through Dual Regulatory Effects on the VEGF/VEGFR Axis,” Theranostics 16 (2026): 1905–1924, 10.7150/thno.122241. 41356185 PMC12680595

[150] M. Pein , J. Insua‐Rodriguez , T. Hongu , et al., “Metastasis‐Initiating Cells Induce and Exploit a Fibroblast Niche to Fuel Malignant Colonization of the Lungs,” Nature Communications 11, no. 1 (2020): 1494, 10.1038/s41467-020-15188-x. PMC7083860 32198421

[151] A. C. Obenauf and J. Massague , “Surviving at a Distance: Organ‐Specific Metastasis,” Trends in Cancer 1 (2015): 76–91, 10.1016/j.trecan.2015.07.009. 28741564 PMC4673677

[152] Y. Li , F. Liu , Q. Cai , et al., “Invasion and Metastasis in Cancer: Molecular Insights and Therapeutic Targets,” Signal Transduction and Targeted Therapy 10, no. 1 (2025): 57, 10.1038/s41392-025-02148-4. 39979279 PMC11842613

[153] R. R. Langley and I. J. Fidler , “Tumor Cell‐Organ Microenvironment Interactions in the Pathogenesis of Cancer Metastasis,” Endocrine Reviews 28, no. 3 (2007): 297–321, 10.1210/er.2006-0027. 17409287

[154] Y. Wang , J. Jia , F. Wang , et al., “Pre‐Metastatic Niche: Formation, Characteristics and Therapeutic Implication,” Signal Transduction and Targeted Therapy 9, no. 1 (2024): 236, 10.1038/s41392-024-01937-7. 39317708 PMC11422510

[155] H. Peinado , H. Zhang , I. R. Matei , et al., “Pre‐Metastatic Niches: Organ‐Specific Homes for Metastases,” Nature Reviews Cancer 17, no. 5 (2017): 302–317, 10.1038/nrc.2017.6. 28303905

[156] Q. Dong , X. Liu , K. Cheng , J. Sheng , J. Kong , and T. Liu , “Pre‐Metastatic Niche Formation in Different Organs Induced by Tumor Extracellular Vesicles,” Frontiers in Cell and Developmental Biology 9 (2021): 733627, 10.3389/fcell.2021.733627. 34616739 PMC8489591

[157] M. Murgai , W. Ju , M. Eason , et al., “KLF4‐Dependent Perivascular Cell Plasticity Mediates Pre‐Metastatic Niche Formation and Metastasis,” Nature Medicine 23, no. 10 (2017): 1176–1190, 10.1038/nm.4400. PMC5724390 28920957

[158] A. E. Paiva , L. Lousado , D. A. P. Guerra , et al., “Pericytes in the Premetastatic Niche,” Cancer Research 78, no. 11 (2018): 2779–2786, 10.1158/0008-5472.CAN-17-3883. 29789421 PMC6044472

[159] A. Hoshino , B. Costa‐Silva , T. L. Shen , et al., “Tumour Exosome Integrins Determine Organotropic Metastasis,” Nature 527, no. 7578 (2015): 329–335, 10.1038/nature15756. 26524530 PMC4788391

[160] L. Patras , L. Shaashua , I. Matei , and D. Lyden , “Immune Determinants of the Pre‐Metastatic Niche,” Cancer Cell 41, no. 3 (2023): 546–572, 10.1016/j.ccell.2023.02.018. 36917952 PMC10170403

[161] B. Costa‐Silva , N. M. Aiello , A. J. Ocean , et al., “Pancreatic Cancer Exosomes Initiate Pre‐Metastatic Niche Formation in the Liver,” Nature Cell Biology 17, no. 6 (2015): 816–826, 10.1038/ncb3169. 25985394 PMC5769922

[162] X. Yang , Y. Zhang , Y. Zhang , et al., “The Key Role of Exosomes on the Pre‐Metastatic Niche Formation in Tumors,” Frontiers in Molecular Biosciences 8 (2021): 703640, 10.3389/fmolb.2021.703640. 34595207 PMC8476876

[163] Y. Li , Y. Zheng , X. Tan , Y. Du , Y. Wei , and S. Liu , “Extracellular Vesicle‐Mediated Pre‐Metastatic Niche Formation via Altering Host Microenvironments,” Frontiers in Immunology 15 (2024): 1367373, 10.3389/fimmu.2024.1367373. 38495881 PMC10940351

[164] L. T. Lyle , P. R. Lockman , C. E. Adkins , et al., “Alterations in Pericyte Subpopulations Are Associated with Elevated Blood–Tumor Barrier Permeability in Experimental Brain Metastasis of Breast Cancer,” Clinical Cancer Research 22, no. 21 (2016): 5287–5299, 10.1158/1078-0432.CCR-15-1836. 27245829 PMC5093086

[165] J. Wang , Z. Cao , X. M. Zhang , et al., “Novel Mechanism of Macrophage‐Mediated Metastasis Revealed in a Zebrafish Model of Tumor Development,” Cancer Research 75, no. 2 (2015): 306–315, 10.1158/0008-5472.CAN-14-2819. 25492861

[166] C. Liu , Y. Zhang , S. Lim , et al., “A Zebrafish Model Discovers a Novel Mechanism of Stromal Fibroblast‐Mediated Cancer Metastasis,” Clinical Cancer Research 23, no. 16 (2017): 4769–4779, 10.1158/1078-0432.CCR-17-0101. 28420724

[167] X. He , X. Yin , J. Wu , et al., “Visualization of Human T Lymphocyte‐Mediated Eradication of Cancer Cells In Vivo,” Proceedings of the National Academy of Sciences 117 (2020): 22910–22919, 10.1073/pnas.2009092117. PMC7502706 32859758

[168] P. Rouhi , L. D. Jensen , Z. Cao , et al., “Hypoxia‐Induced Metastasis Model in Embryonic Zebrafish,” Nature Protocols 5, no. 12 (2010): 1911–1918, 10.1038/nprot.2010.150. 21127485

